# Impaired coupling of local and global functional feedbacks underlies abnormal synchronization and negative symptoms of schizophrenia

**DOI:** 10.1186/1752-0509-7-30

**Published:** 2013-04-10

**Authors:** Kyungchul Noh, Kyung Soon Shin, Dongkwan Shin, Jae Yeon Hwang, June Sic Kim, Joon Hwan Jang, Chun Kee Chung, Jun Soo Kwon, Kwang-Hyun Cho

**Affiliations:** 1 Department of Bio and Brain Engineering, Korea Advanced Institute of Science and Technology (KAIST), Daejeon, Republic of Korea; 2 Clinical Cognitive Neuroscience Center, Neuroscience Institute, SNU-MRC, Seoul, Republic of Korea; 3 MEG Center, Department of Neurosurgery, Seoul National University College of Medicine, Seoul, Republic of Korea; 4 Department of Psychiatry, SMG-SNU Boramae Medical Center, Seoul, Republic of Korea; 5 Department of Psychiatry, Seoul National University College of Medicine, Seoul, Republic of Korea; 6 Department of Brain and Cognitive Sciences, World Class University Program, College of Natural Sciences, Seoul National University, Seoul, Republic of Korea

**Keywords:** Abnormal synchrony, Network topology, Coupled feedback, Schizophrenia, Negative symptom

## Abstract

**Background:**

Abnormal synchronization of brain oscillations is found to be associated with various core symptoms of schizophrenia. However, the underlying mechanism of this association remains yet to be elucidated.

**Results:**

In this study, we found that coupled local and global feedback (CLGF) circuits in the cortical functional network are related to the abnormal synchronization and also correlated to the negative symptom of schizophrenia. Analysis of the magnetoencephalography data obtained from patients with chronic schizophrenia during rest revealed an increase in beta band synchronization and a reduction in gamma band power compared to healthy controls. Using a feedback identification method based on non-causal impulse responses, we constructed functional feedback networks and found that CLGF circuits were significantly reduced in schizophrenia. From computational analysis on the basis of the Wilson-Cowan model, we unraveled that the CLGF circuits are critically involved in the abnormal synchronization and the dynamical switching between beta and gamma bands power in schizophrenia. Moreover, we found that the abundance of CLGF circuits was negatively correlated with the development of negative symptoms of schizophrenia, suggesting that the negative symptom is closely related to the impairment of this circuit.

**Conclusions:**

Our study implicates that patients with schizophrenia might have the impaired coupling of inter- and intra-regional functional feedbacks and that the CLGF circuit might serve as a critical bridge between abnormal synchronization and the negative symptoms of schizophrenia.

## Background

Schizophrenia is one of psychotic mental disorders and is characterized by various symptoms including hallucination, thought disorder, absence of behavior, and lack of motivation. These psychopathologies usually emerge as abnormalities in oscillatory activities of neurons and their synchronizations [1,2]. A number of studies using magnetoencephalography (MEG) and electroencephalography (EEG) have demonstrated that symptoms of schizophrenia might be related to aberrantly enhanced or reduced synchrony of oscillations in high (beta and gamma) frequency bands during cognitive tasks or at rest [3-8]. These findings suggest that impaired synchronization of beta- and gamma-band oscillations could underlie the dysfunctions in cortical communications in schizophrenia. However, it is still unclear what the underlying mechanism is for the association between abnormal synchronized oscillations and the core symptoms of schizophrenia.

Anatomical investigations have shown that interregional connectivity is highly reciprocal [9,10] and such recurrent connections have an important role in modulating visual and auditory sensory stimuli [11,12]. Dysfunction in such a feedback structure can produce abnormal synchronization under pathological conditions. Several computational studies have shown that cortical feedback connections are critical to promote the synchronization among distributed neuronal networks [13-15]. A feedback connection is crucial to generate biological oscillations, and feedback loops coupling local oscillators contribute to inducing and modulating their synchronization. Biological experiments showed that feedback circuits are ubiquitously found in a variety of biological subjects [16-18] and theoretical studies proved the relationship between feedback loop and synchronized oscillation [19,20]. On the basis of these findings, we hypothesize that the impairment of functional feedbacks among distributed brain areas may underlie the abnormal synchronization in schizophrenia and provide a bridge between abnormal synchrony and the symptoms of schizophrenia.

We investigate this hypothesis by constructing and analyzing the functional feedback networks of patients with schizophrenia and normal controls during rest on the basis of MEG measurements and feedback identification. Resting state, a condition in the absence of stimulus or cognitive task, is important to understand the spontaneous oscillation of brain activity and is considered as a baseline condition in many neuroimaging studies [21-24]. The analysis of power spectrum and phase synchronization showed an abnormally reduced gamma band power and an increased beta band phase synchrony in patients group. From the inferred functional feedback network, we found that coupled local and global feedback (CLGF) circuit is differently enriched in both groups. Through computational analysis based on the Wilson-Cowan model, we found that this circuit is critically involved in the abnormal neural oscillations and synchronization in schizophrenia and that this circuit mediates the dynamical switching between beta and gamma bands power. In addition, we found that the abundance of this circuit is strongly correlated with the negative symptoms of schizophrenia, suggesting that the negative symptom is closely related to the impairment of this circuit. Taken together, we suggest that the CLGF circuit might be a critical determinant of abnormal neural oscillations and synchrony in schizophrenia and that the impairment of this circuit accelerates the negative symptoms of schizophrenia. This study is to our knowledge the first attempt to identify the underlying mechanism, at a system-level, that bridges the abnormal synchronized oscillations and core symptoms of schizophrenia in consideration of the functional feedback networks of the whole cortical brain area.

## Methods

### Participants and clinical assessments

Healthy control subjects and schizophrenia patients overlapped with those subjects in our previous study [25] but not identical. This study protocol was approved by the Institutional Review Board of Seoul National University Hospital (SNUH) and the study was conducted at the MEG center in SNUH. All participants gave an informed consent form to their participation.

As shown in Table 1, 15 patients with schizophrenia and 17 healthy comparison subjects were matched for their age, sex, and handedness for participation in the study. The schizophrenia subjects who were diagnosed and fulfilled Diagnostic and Statistical Manual of Mental Disorders (Fourth Edition; DSM-IV) were assessed with Positive and Negative Syndrome Scale (PANSS) at the baseline MEG assessment [26] and the positive symptom score, negative symptom score, and general psychopathology score were calculated. Among 15 patients with schizophrenia, one was not taking any medication at the MEG assessment. Healthy controls were screened by the Structured Clinical Interview for DSM-IV Non-Patient version (SCID-NP). Healthy control subjects were applied with an additional exclusion criterion of any first- or second-degree biological relatives with a lifetime history of a psychotic disorder. Exclusion criteria for all participants included any lifetime diagnosis of psychotic illness, substance dependence, a neurological disease, a history of head injury or a full-scale IQ estimate of less than 70.

**Table 1:** Demographic and clinical assessment

	**Healthy controls**	**Schizophrenia**	**Analysis**		
	**(n=17)**	**(n=15)**			
	**Mean ± SD**	**Mean ± SD**	**T or χ**^**2**^	**df**	***P***
Male/Female	11/6	12/3	0.95	1	0.348
Age (yrs)	22.06 ± 2.11	23.80 ± 4.60	-1.35	1	0.194
Education (yrs)	14.06 ± 1.20	13.27 ± 2.25	1.22	1	0.236
Handedness	11.35 ± 1.69	10.53 ± 2.59	1.05	1	0.307
IQ	108.35 ± 17.26	103.33 ± 9.90	1.02	1	0.316
PANSS		56.47 ± 13.14			
Duration of illness (yrs)		6.89 ± 3.14			

### MEG Data acquisition and preprocessing

A 306 MEG system (Elekta Neuromag Oy, Helsinki, Finland) was used to record MEG activities. The MEG signals were band-pass filtered (0.1 to 200 Hz) and digitized at a 1000 Hz sampling rate. All subjects were instructed to focus on crossed figure at the screen to prevent random eye movements. During recording, no external stimulus was given. After recording, noise reduction and compensation of head movement were carried out using tSSS algorithm [27]. Resting condition was recorded with open eyes during 120 seconds. Data were visually inspected to reject epochs with unnecessary noise, such as eye movement and muscle artifacts. A notch filter was used to remove artifacts of 60 Hz and its harmonics (120 and 180 Hz). MEG data were imported into MATLAB 7.8.0 (R2009a) in Windows for data processing. Using 102 magnetometers, 80 seconds time points with a 1000 Hz sampling rate were considered for all analysis in this study.

### Time-frequency and power analysis

We considered 13~80 Hz frequency components of the MEG data corresponding to beta and gamma bands and carried out time-frequency analysis of MEG signals using complex Morlet wavelet transform [28]. The width of the wavelet was defined as 7 that encompasses at least one full sinusoidal cycle for any particular frequency [29]. The absolute values of the resulting transforms from 102 magnetometers were averaged. Mean power spectrum was calculated on the basis of the average time-frequency analysis result.

### Beta-gamma power switching

**Figure 1:**
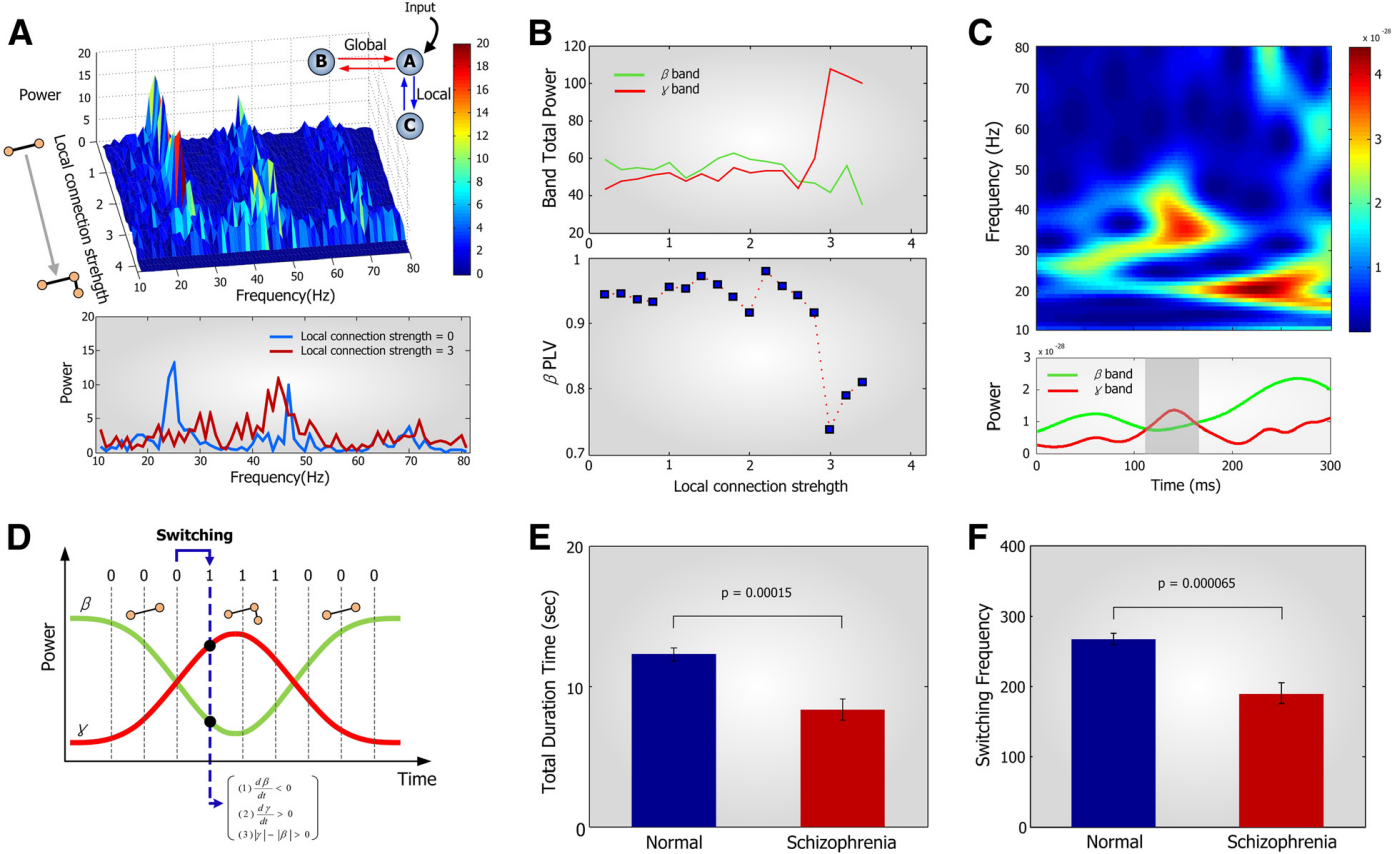
**Modeling of the coupled local and global feedback circuits and the dynamical switching of beta-gamma power. **(**A**) Frequency spectrum of the coupled local and global feedback circuits along with the varying local connection strength. Schematic diagram of the local and global feedback circuit was shown. Bottom figure represents the frequency spectrum in the case of local connection strength of 0 and 3, which correspond to 2-node positive feedback and the CLGF circuit, respectively. (**B**) (*Top*) Changes in the gamma power of oscillators *a *and *b *along with the increase of local connection strength. (*Bottom*) Changes in beta band phase locking value between oscillators *a *and *b*. (**C**) (*Top*) Representative example of the beta-gamma power switching in MEG data. Time-frequency pattern of beta and gamma bands power. (*Bottom*) Averaged power plot of beta and gamma bands. (**D**) Schematic representation of beta-gamma power switching. In each 1 *ms *time step, 0 was assigned for no switching and 1 for beta-gamma power switching. The duration of beta-gamma switching was determined by summing all these values, and the frequency of switching events was determined by counting the number of time steps changing from 0 to 1. (**E**) The duration and (**F**) the frequency of beta-gamma power switching in both normal and schizophrenia patients groups. Both measurements are significantly reduced in the patients group (p = 0.00015 and p = 0.000065). Bars and vertical lines indicate mean and standard errors, respectively.

Beta-gamma switching was investigated based on the result of time-frequency analysis. We used three conditions for switching measurement: (*i*) negative value of discrete-time derivative of beta power (*dβ* / *dt* < 0), (*ii*) positive value of discrete-time derivative of gamma power (*dγ | dt > 0*), and (*iii*) greater gamma power than beta power (*γ* - *β* > 0). For the triggering of switching, these three conditions must be met simultaneously. We set the switching value to 1 until the absolute beta band power is greater than that of gamma band. Otherwise, we set it to 0. So, the switching value was set to 0 or 1 at each 1 *ms* time step based on three conditions described above (Figure 1D). Among 80 seconds of the resting period (total 80,000 time points), we summed all values to obtain the total duration of beta-gamma power switching. To obtain the frequency of beta-gamma power switching, we counted the number of time steps changing from 0 to 1 over the period of resting state.

### Phase synchronization

MEG signals were filtered into beta (13 ~ 30 Hz) and gamma (30 ~ 80 Hz) bands by using ‘eegfilt’ in EEGLAB toolbox [30]. We analyzed the phase synchrony of beta- and gamma-band signals using a sliding-window approach with the window size of 1000 *ms* and the sliding size of 200 *ms*. Hilbert transform was applied to get phase information in both beta and gamma bands. We found phase differences between all pairs of magnetometer sensors and considered phase locking value (PLV) as a synchronization index, $\gamma_{n,m}=\sqrt{<\text{sin}\varphi_{n,m}\left(t\right){>}^{2}+<\cos\varphi_{n,m}\left(t\right){>}^{2}}$, which ranges from 0 for no synchronization to 1 for perfect synchronization [31] where *φ*_*n*,*m*_ represents the phase difference between signal *n* and *m*. This index is useful in that it measures how the relative phase is distributed over a unit circle. We calculated the PLV values for all possible combinations of magnetometer sensors and averaged them for each subject.

### Feedback identification based on impulse responses

**Figure 2:**
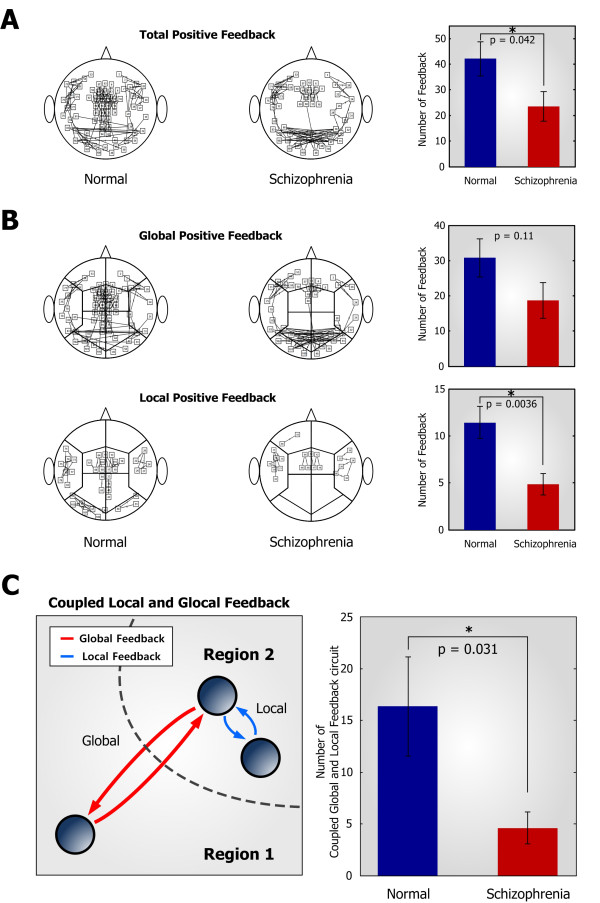
**Comparison of functional feedback networks and coupled local and global feedback circuits between two groups. **(**A**) (*Left *and *Middle*) Total number of positive feedback connections in the normal and schizophrenia patients groups, and (*Right*) bar plot of the total number of positive feedback connections. Patients group exhibits the reduced number of positive feedback connections (p = 0.042). (**B**) (*Top row*) The number of global and (*Bottom row*) local positive feedback connections in the normal and schizophrenia patients groups. The reduced number of local positive feedback connections can be seen in the patients group (p = 0.0036). (**C**) (*Left*) Coupled local and global feedback circuits existing in the cortical functional network and (*Right*) statistical comparison of the number of this circuit in the normal and schizophrenia patients groups. Coupled local and global feedback circuits are reduced in the patients group (p = 0.031). Bars and vertical lines denote mean and standard errors, respectively.

To identify the functional connectivities of the MEG sensor network, we applied the feedback identification method based on impulse responses [32,33]. This method uses an extended concept of the impulse response function including non-causal impulse response components. Both positive and negative feedback relationships can be identified by comparing impulse responses of causal and non-causal components (Additional file 1: Figure S1). To identify feedback relationships at a high resolution, 20 *sec* time window was used and both positive and negative feedbacks were identified in four windows that were not overlapped with each other (4 × 20 *sec* = 80 *sec*) for each subject in a group. So, for each individual subject, four networks were inferred and feedback connections were selected if and only if identical feedback connections were consistently found in all networks. The same procedure was repetitively applied to all subjects in two groups. The topological visualization of a network in Figure 2 shows the averaged network in each group. We used Mavisto v2.7.0 software for topological visualization of a network.

### Network simulations based on the Wilson-Cowan model

The Wilson-Cowan model [34] was employed in this study for simulation analysis of CLGF circuits and it is described as follows:

$$\frac{\mathrm{dE}}{\mathrm{dt}}=-\mathrm{\alpha E}+\beta_{E}\left(1-\mathrm{rE}\right)\cdot f\left(s_{E}\right),$$

$$\frac{\mathrm{dI}}{\mathrm{dt}}=-\mathrm{\alpha I}+\beta_{I}\left(1-\mathrm{rI}\right)\cdot f\left(s_{I}\right),$$

where *α* represents the natural decay, *β*_*E*_ and *β*_*I*_ are the maximal firing rates of excitatory and inhibitory populations, respectively, *r* is the refractory constant, and both *s*_*E*_ and *s*_*I*_ denote the total incoming inputs that are represented as *s*_*E*_ = *ω*_*EE*_*E* - *ω*_*EI*_*I* + *P* and *s*_*I*_ = *ω*_*IE*_*E* - *ω*_*II*_*I* + *Q*, respectively. In the input equations, the internal terms, (*ω*_*EE*_, *ω*_*EI*_, *ω*_*IE*_, *ω*_*II*_), denote synaptic weights between excitatory-excitatory, excitatory-inhibitory, inhibitory-excitatory, and inhibitory-inhibitory populations, and *P* and *Q* indicate the external constant inputs to excitatory and inhibitory neurons, respectively. Here *f(s)* is a sigmoid response function defined as *f*(*s*) = 1/[1 + exp(-*a*(*s* - *θ*))] - 1/[1 + exp(*aθ*)] where *a* determines the maximum slope of the response function and *θ* is the neural threshold. For this oscillator model, to find a stable limit cycle with a frequency of at least 80 Hz for varying external excitatory inputs, we obtained the following parameter estimates: *α* = 0.35, *β*_*E*_ = *β*_*I*_ =1, *r* = 0.65, *a*_*E*_ = 1.3, *a*_*I*_ = 2, *θ*_*E*_ = 4.5, *θ*_*I*_ = 3.5, ω_*EE*_ = 16, ω_*EI*_ = 15, ω_*IE*_ = 12, and ω_*II*_ = 3. The inhibitory input Q was set to 0 and the excitatory input *P* was varied from 0 to 5. With these parameter estimates, we have constructed the model of CLGF circuit as follows:

$$\begin{array}{l} \frac{dE_{a}}{\mathrm{dt}}=-\alpha E_{a}+\beta_{E}\left(1-rE_{a}\right)\cdot f(s_{E,\text{abc}})\\ \frac{dI_{a}}{\mathrm{dt}}=-\alpha I_{a}+\beta_{I}\left(1-rI_{a}\right)\cdot f(s_{I,\text{abc}})\\ \frac{dE_{b}}{\mathrm{dt}}=-\alpha E_{b}+\beta_{E}\left(1-rE_{b}\right)\cdot f(s_{E,\mathrm{ba}})\\ \frac{dI_{b}}{\mathrm{dt}}=-\alpha I_{b}+\beta_{I}\left(1-rI_{b}\right)\cdot f(s_{I,\mathrm{ba}})\\ \frac{dE_{c}}{\mathrm{dt}}=-\alpha E_{c}+\beta_{E}\left(1-rE_{c}\right)\cdot f(s_{E,\mathrm{ca}})\\ \frac{dI_{c}}{\mathrm{dt}}=-\alpha I_{c}+\beta_{I}\left(1-rI_{c}\right)\cdot f(s_{I,\mathrm{ca}}), \end{array}$$

where

$$\begin{array}{l} s_{E,\text{abc}}\left(t\right)=\omega_{\mathrm{EE}}E_{a}(t)-\omega_{\mathrm{EI}}I_{a}(t)+\omega_{\mathrm{ab}}E_{b}(t-\tau_{\text{global}})\\ \;+\omega_{\mathrm{ac}}E_{c}(t-\tau_{\text{local}})+P\\ s_{I,\text{abc}}\left(t\right)=\omega_{\mathrm{IE}}E_{a}(t)-\omega_{\mathrm{II}}I_{a}(t)+Q\\ s_{E,\mathrm{bc}}\left(t\right)=\omega_{\mathrm{EE}}E_{b}(t)-\omega_{\mathrm{EI}}I_{b}(t)+\omega_{\mathrm{ba}}E_{a}(t-\tau_{\text{global}})\\ s_{I,\mathrm{bc}}\left(t\right)=\omega_{\mathrm{IE}}E_{b}(t)-\omega_{\mathrm{II}}I_{b}(t)\\ s_{E,\mathrm{ca}}\left(t\right)=\omega_{\mathrm{EE}}E_{c}(t)-\omega_{\mathrm{EI}}I_{c}(t)+\omega_{\mathrm{ca}}E_{a}(t-\tau_{\text{local}})\\ s_{I,\mathrm{ca}}\left(t\right)=\omega_{\mathrm{IE}}E_{c}(t)-\omega_{\mathrm{II}}I_{c}(t). \end{array}$$

The details on the structure and variables of this CLGF circuit are described in Additional file 1: Figure S2C. The global connection strength between oscillators *a* and *b* (*ω*_*ab*_ and *ω*_*ba*_), and the local connection strength between oscillators *a* and *c* (*ω*_*ac*_ and *ω*_*ca*_) were set as *ω*_*ab*_ = *ω*_*ba*_ and *ω*_*ac*_*=*_*ca*_, respectively, and both were explored over a range from 0 to 4. These delayed differential equations were solved numerically using dde23 in MATLAB 7.8.0 (R2009a).

The simulation procedures were as follows: Initially, the constant external excitatory input ranging from 0 to 5 was applied to the excitatory population in oscillator *a* (Additional file 1: Figure S2A *Left*). This oscillator exhibited an oscillation frequency ranging from 80 to 150 Hz for input values from 1.2 to 3, and no oscillation or hyper-excitation otherwise (Additional file 1: Figure S2A *Right*). We selected the input value so that the oscillator *a* exhibits around 100 Hz frequency oscillations and then we connected the oscillator *b* to the oscillator *a* through positive feedback connection (Additional file 1: Figure S2B *Left*). As the connection strength was varied from 0 to 4, this structure exhibited additional frequency components generated by the positive feedback between oscillators *a* and *b* for the connection strength of 3 (Additional file 1: Figure S2B *Right*). We ignored any hyper-excitatory oscillation which is the typical characteristic of a highly activated positive feedback loop. In the next, we added the third oscillator *c* to the oscillator *a* through positive feedback. The resulting frequency spectrum of this structure is displayed in Figure 1A. The total beta and gamma band powers of the CLGF circuit were calculated by summing each frequency band power. Moreover, beta band phase synchronization between oscillators *a* and *b* was investigated on the basis of the phase locking value.

### Statistical analysis

A two-tailed *t*-test was carried out to examine the group differences of power, phase synchronization, the number of positive feedbacks, the number of CLGF circuits, and the switching of beta-gamma power. The *t*-test was also used to compare the demographic and the clinical assessments between groups. Pearson correlation coefficients were calculated using MATLAB 7.8.0 (R2009a) to examine the correlations between the number of CLGF circuits and the clinical symptoms (PANSS) within the schizophrenia group. The α-level was set to 0.05 for all statistical tests.

## Results

### Schizophrenia patients showed an abnormally reduced gamma power and increased beta phase synchronization

**Figure 3:**
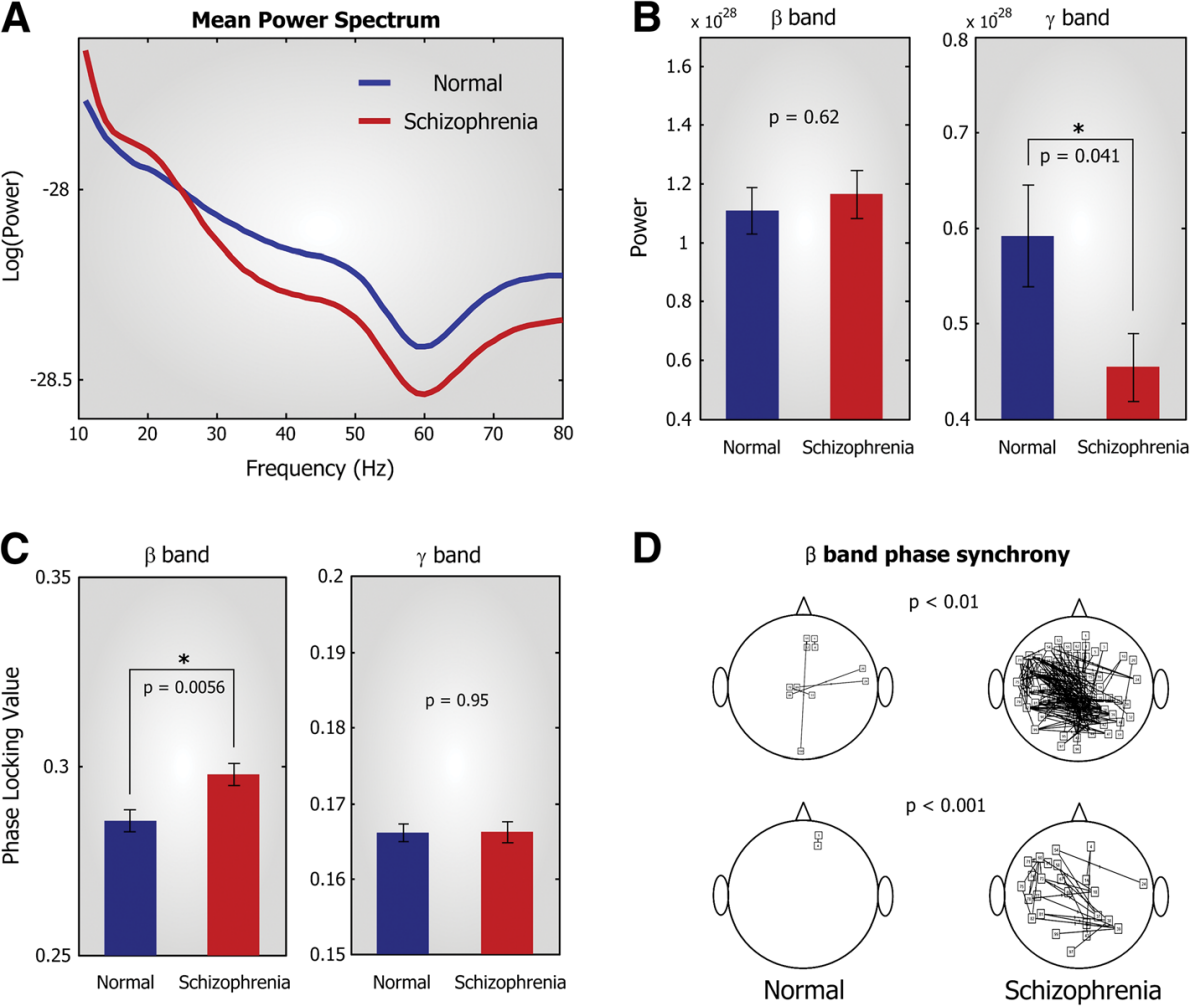
**Spectral power and phase synchronization of beta and gamma bands. **(**A**) Averaged power spectrum of normal (blue) and schizophrenia patients (red) group. Patients group exhibits reduced mean power above 25 Hz frequency range. (**B**) Bar plot of beta and gamma band power in normal and schizophrenia patients groups. Gamma band was significantly reduced in the patients group (p = 0.041). (**C**) Bar plot of beta and gamma band phase synchronization in the normal and schizophrenia patients groups. Beta band phase locking value (PLV) increased in the schizophrenia patients group (p = 0.0056). Bars and vertical lines indicate mean and standard errors, respectively. (**D**) Topological comparison of beta band phase locking value between two groups. Each edge represents significantly stronger beta band phase locking value than the other group based on p-value of 0.01 (*Top*) and 0.001 (*Bottom*).

We carried out frequency spectrum analysis using wavelet transform to both healthy controls and schizophrenia patients. Over 80 seconds of data, we quantified the spectral power of each frequency band and averaged it with respect to time (see Methods for details). The results showed an increased beta (13 ~ 30 Hz) band power and a decreased gamma (31 ~ 80 Hz) band power in patients group (Figure 3A). Statistical analysis revealed that the gamma band power was significantly reduced in schizophrenia patients relative to healthy controls (p = 0.041) (Figure 3B) whereas the beta band power was not significantly different between the two groups (p = 0.62). To investigate whether such abnormal oscillations are also accompanied by an abnormality in inter-regional synchronization, we calculated the phase locking value (PLV) across all pairs of MEG sensors as a measure of synchronization (see Methods for mathematical description). The PLV ranges from 0 to 1 indicating no and perfect synchronization, respectively. Relative to healthy controls, schizophrenia patients showed a profound increase in beta band phase synchronization (p = 0.0056) while there was no significant difference between the two groups in gamma band (p = 0.95) (Figure 3C). Figure 3D illustrates a topographic difference in beta band phase synchronization between groups, where each connection represents significantly higher PLV of one group than that of the other group (Top; p < 0.01, Bottom; p < 0.001). In patients with schizophrenia, the connectivity of beta band phase synchronization was significantly increased over most of the brain areas relative to controls. We repeated the same procedure with the threshold p-value of 0.01 and 0.001, respectively, and obtained consistent distribution patterns of connectivity (Figure 3D). Taken together, our results indicate that schizophrenia patients had a decreased gamma band power and an increased beta band phase synchronization. To investigate the underlying mechanism of these abnormalities, we focused on the cortical functional network of normal and schizophrenia patients and the analysis results are presented in the next section.

### Patients with schizophrenia have sparse CLGF circuits

The frequency of synchronized oscillations is correlated with the distance over which synchronization occurs. It has been proposed that the gamma band activity is associated with short-distance synchronization whereas the beta band activity supports long-distance synchronization [15], implying that deficits in neural oscillations and synchronization might be accompanied with functional disconnection between brain areas as well as anatomical connectivity. To compare the topological difference between normal controls and patients with schizophrenia in their functional connectivities, we inferred both positive and negative feedback connections among 102 MEG magnetometer sensors by using the impulse response feedback identification method [32]. Results showed that the total number of positive feedbacks was significantly reduced in schizophrenia patients group (p = 0.042) (Figure 2A) while there was no significant difference in negative feedback (Additional file 1: Figure S3A). Moreover, both groups exhibited relatively a less number of negative connections (Additional file 1: Figure S3B), indicating that negative feedback does not much contribute to the abnormalities of oscillation and synchronization. To investigate the spatial organization of functional feedback connectivities, we classified magnetometer sensors into 10 cortical subregions including pre-frontal, middle-frontal, temporal, parietal, and occipital regions in left and right hemispheres (Figure 2B). From the fact that the inter-regional conduction delay is longer than intra-regional delay [35,36], we divided the positive feedback connections into two types: (*i*) global positive feedback between subregions having a long time delay, and (*ii*) local positive feedback within a subregion having a short time delay. While the number of global positive feedbacks was not significantly different between the two groups (p = 0.11) (Figure 2B, *Top row*), the number of local positive feedbacks was reduced in patients group (p = 0.0036) (Figure 2B, *Bottom row*).

From our results, we found that schizophrenia patients have sparse positive feedbacks in their cortical network although they show enhanced phase synchronization of a beta band activity. These results seem to be incompatible with the commonly observed synchronization property that a larger coupling strength between two oscillators tends to induce a higher degree of synchronization [19]. Therefore, we presumed that the abnormal synchronization in schizophrenia is attributed not only to the abundance of feedback connections but also to the detailed coupling patterns of feedback connections. For instance, if oscillators are coupled with each other through positive feedback connections having different conduction delays, oscillators are not synchronized properly [37] even though they are coupled through a large number of feedback connections. On the basis of this, we further investigated a coupled structure of short and long time delay connections that constitute a coupled local and global feedback (CLGF) circuit in the referred functional feedback networks (Figure 2C, *Left*). In patients with schizophrenia, the number of CLGF circuits was significantly reduced relative to that of healthy controls (p = 0.031) (Figure 2C, *Right*). In addition, we constructed functional networks based on partial correlation and mutual information to explore the changes in functional connectivity of schizophrenia. The results showed that the functional network of schizophrenia patients have lower coupled local and global connectivity than that of controls, which is consistent with the results of the functional feedback networks inferred by applying the impulse response method (see Additional file 1: Figure S4 and S5, and supporting methods in Additional file 2). These results suggest that deficits in the CLGF circuits might be responsible for the decreased gamma band power and the increased beta band phase synchronization in schizophrenia. Hence, detailed computational analysis has been carried out to elucidate the influence of such structural impairments on the oscillatory activities and synchronization.

### Deficits in the CLGF circuits induce the impairment of beta-gamma power switching

We carried out neural network simulations of the CLGF circuits using the Wilson-Cowan model [34] which is widely used to describe a neuronal ensemble as an oscillator model composed of excitatory and inhibitory neurons (see Methods for details). To investigate the changes in the frequency spectrum of the CLGF circuits with respect to varying connection strengths, coupled oscillators were considered as shown in Figure 1A where an oscillator *a* is coupled with oscillators *b* and *c* through feedback loops with long (21 *ms*) and short (6 *ms*) time delays, respectively. While the oscillator *a* coupled with the oscillator *b* through a global feedback loop exhibited mostly beta band oscillations (local connection strength = 0), the oscillator *a* coupled with both oscillators *b* and *c* through global and local feedback loops showed an increased gamma oscillation with a reduced beta oscillation (local connection strength = 3) as shown in Figure 1A (*Bottom*). In the case of weak local connection strength, the beta band power was dominant over the gamma band power although we did not remove the artifacts of beta harmonics in the gamma band (Figure 1B, *Top*). As the coupling of local and global feedbacks gets strengthened beyond the threshold of local connection strength, the gamma band power was abruptly increased and became dominant over the beta band. This result confirms that deficits of the CLGF circuits in a cortical network are responsible for the decreased gamma band power in patients with schizophrenia. Moreover, beta band phase synchronization was suddenly decreased in the case of strong local connection (Figure 1B, *Bottom*), suggesting that the increased beta band synchronization might also result from the deficit of the CLGF circuits in patients with schizophrenia. These properties were well conserved even when global and local time delays were varied over the ranges of 15–25 *ms* and 6–10 *ms*, respectively (Additional file 1: Figure S6). Note here that we adopted the inter-regional conduction delay in the range of 15~25 *ms* for global feedbacks and the intra-regional delay in the range of 6~10 *ms* for local feedbacks based on previous experiments [35,36,38-40].

To explore how often and how long such beta to gamma power shift occurs in MEG data, time-frequency analysis based on the wavelet transform was carried out for both groups. Figure 1C shows an example of the beta-gamma power shift in a healthy subject. We found that there exists a period where the gamma power increased, but, simultaneously, the beta power decreased. According to our simulation results, the CLGF circuits might be activated during this period. To compare the proportion of this switching period in both groups, we measured the duration in which the power switching between beta and gamma occurred. In each 1 *ms* time step, we assigned 1 when temporal profiles of beta and gamma powers satisfy three conditions, (*d β* / *dt* < 0, *d γ* /*dt* > 0, and *γ* -*β* > 0), and kept this value until the beta power becomes larger than the gamma power (Figure 1D) (see Methods for details). We found that schizophrenia patients showed a significant reduction of the duration involved in the beta-gamma switching (p = 0.00015), which indicates that the CLGF circuits are deactivated in schizophrenia (Figure 1E). We further found that the frequency of switching events was also significantly reduced in the patients group (p = 0.000065) (Figure 1E) and this suggests that the impairment of dynamical switching between beta and gamma bands is also an important characteristic of schizophrenia in addition to the abnormal gamma power and beta synchronization. Taken together, deficits in the CLGF circuits in schizophrenia might be critically involved in the abnormalities in neural oscillations, synchronization, and temporal switching of frequency bands.

### The CLGF circuit is inversely correlated with the negative symptoms of schizophrenia

**Figure 4:**
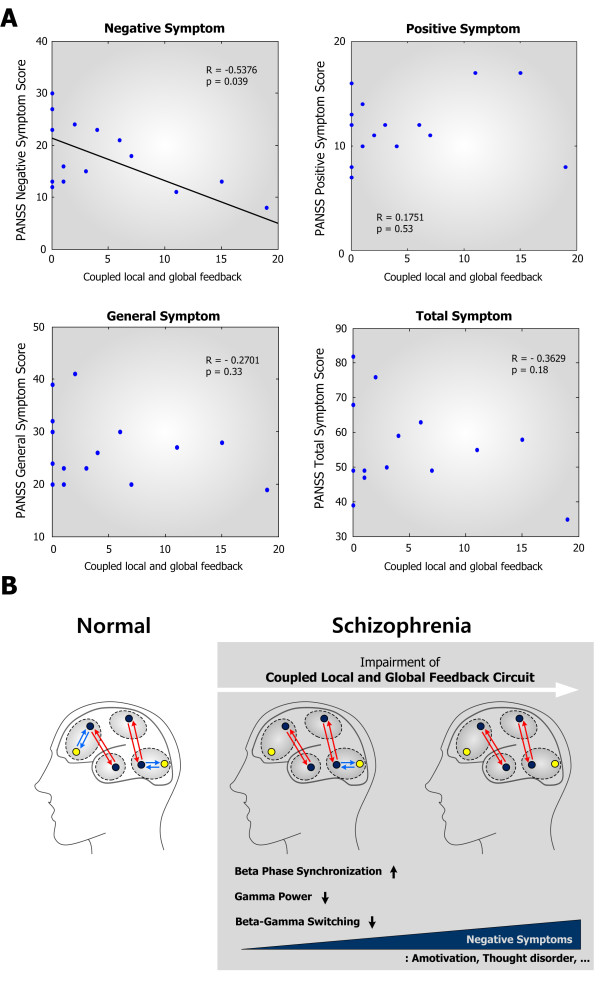
**Correlation of the CLGF circuit with clinical variables. **(**A**) PANSS negative symptom score was correlated with the number of CLGF circuits in the functional feedback networks of schizophrenia (R = -0.5376, p = 0.039) whereas PANSS positive, negative, general, and total symptom scores did not show significant correlations. (**B**) Schematic diagram summarizing this study. Relative to healthy controls, schizophrenia patients exhibit reduced interaction between interregional and intraregional connections, leading to impairment of CLGF circuit. Deficits in the coupled feedback circuit result in abnormal oscillations such as reduced gamma power and increased beta synchronization in the schizophrenia patients group. Reduced beta-gamma power switching might also be attributed to the impairment of CLGF circuit. Schizophrenia patients exhibited that PANSS negative symptom score is inversely proportional to the number of CLGF circuits. This suggests that negative symptoms of schizophrenia is also involved in the disconnectivity of coupled cortical connections.

In the foregoing results, we have shown that the CLGF circuit might be crucial for the abnormal neural oscillations and synchronization in schizophrenia. A question then arises as to whether this circuit is also correlated with the symptoms of schizophrenia. To investigate the significance of CLGF circuit in a clinical dimension, Pearson's correlation analysis was applied to examine the correlation between the number of CLGF circuits and PANSS (Positive and Negative Syndrome Scale) scores of schizophrenia. PANSS positive, negative, general, and total symptom scores were applied to investigate the correlation with the CLGF circuits. Results showed that PANSS negative symptom score was inversely correlated with the abundance of CLGF circuit (p = 0.039) in a significant manner whereas PANSS positive, general, and total symptom scores did not show any significant correlation (Figure 4A). Taken together, the CLGF circuit is negatively correlated with the negative symptoms of schizophrenia, which suggests that the CLGF circuit in cortical functional network might be responsible for the critical mechanism that bridges phenomenological characteristics and the symptoms of schizophrenia.

## Discussion

Negative symptoms of schizophrenia are the most prominent factor that contributes to the functional impairment of patients. So, rather than positive symptoms such as hallucination and delusion, negative symptoms have been suggested as the core of the illness [41]. In this study, we found that the CLGF circuit in cortical functional networks is closely related to the negative symptoms of schizophrenia (Figure 4B). Patients with schizophrenia were characterized by the decreased gamma band power and increased beta band synchronization relative to healthy controls, and network model simulations revealed that such abnormalities resulted from deficits in the CLGF circuit. Moreover, the dynamical switching between beta and gamma bands power in patients was reduced, which might also be induced by the impairment of local feedback connections in the CLGF circuit. Therefore, we infer that patients with schizophrenia might have impaired coupling of inter- and intra-regional functional feedbacks and that the CLGF circuit is a crucial bridge between abnormal synchronization and the negative symptoms of schizophrenia.

It is known that synchronized oscillation is mostly caused by the feedback interaction between oscillators [19,42]. So, the goal of our network inference was to identify such feedback connections among all 102 magnetometer sensors of MEG rather than to infer functional connections that are commonly obtained by correlation or mutual information methods [43-45]. For this purpose, we employed the impulse response method as it is particularly advantageous in dealing with a large network owing to its efficient computational time compared to other methods such as Granger causality or Bayesian network inference methods [42,46-48].

Patients with schizophrenia in our study showed three different phenomenological characteristics compared to healthy controls: (*i*) a reduced gamma band power, (*ii*) increased beta band phase synchronization, and (*iii*) reduced beta-gamma power switching. Consistent with our result, the gamma power reduction in schizophrenia patients under resting condition have been demonstrated in several previous studies [49,50] and the patients were considered to experience the resting condition in a very different way compared to healthy controls. Enhanced beta band oscillation and synchronization were known to be associated with the suppression of a response during the cued choice reaction task [51], which suggests that the increased beta band synchronization in our results might be related to the inability of patients in the preparation for future events. Interestingly, there was a hypothetical study which suggested that abnormally increased beta band synchronization might be induced during a resting state by the rest-rest self interaction in the brain of schizophrenia patients [52]. In addition to the abnormalities in gamma power and beta phase synchronization, the temporal switching between beta and gamma bands power in our results can also be understood in the same context. It is well known that brain frequency bands such as beta or gamma are relevant to the corresponding brain functions. However, little is known about the dynamical interaction between such frequency bands. In this study, our simulation results demonstrated that the CLGF circuit is responsible for the dynamical switching mechanism, suggesting that deficits in this circuit might induce the impairment of beta-gamma switching in schizophrenia. Abnormal temporal dynamics of cortical networks may result in impairments in synaptic plasticity, and such aberrant neurodevelopment has been observed in schizophrenia [53]. Impaired plasticity may lead to difficulties in flexible coordination of distributed brain processes and rapid responses when a task is given. Therefore, schizophrenia patients with reduced beta-gamma band switching are likely to have cognitive deficits due to impaired dynamic interactions between multiple brain regions. Taken together, we suggest that the temporal switching across different frequency bands as well as the abnormal power and synchrony might be important factors characterizing schizophrenia although further studies including both resting and cognitive task conditions are required to clarify the role of temporal switching among frequency bands.

## Conclusions

A brain is inherently a dynamical system that continuously reorganizes functional connections within and between brain regions [54-57]. In the present study, we identified a fundamental network structure motif, the CLGF circuit, responsible for the abnormalities in neural oscillations, synchronization, and temporal switching of frequency bands in schizophrenia. Functional disconnections of this circuit may disturb flexible communications between brain regions, hence eventually contributing to the pathogenesis of schizophrenia as shown in our result that the abundance of CLGF circuit is negatively correlated with the negative symptoms of schizophrenia. Therefore, we conclude that altered functional feedbacks, such as the CLGF circuit, serves as a critical bridge between phenomenological properties and the psychotic symptoms of schizophrenia. Over the past decade, a number of studies using functional brain imaging techniques have attempted to reveal the fundamental mechanism that underlies the association of impaired neural oscillations and synchronization with core symptoms of schizophrenia. Most of the studies, however, have dealt with functional connectivity of a whole brain using simple correlation methods or effective connectivity of a local brain network with only few regions. Here, we introduced a feedback identification method based on non-causal impulse response to construct a functional feedback network covering the whole cortical brain regions, and this enabled us to project a cortical network to a more precise cortico-cortical interaction map. In the future, a combined study with anatomical connectivity will provide further insights into the underlying mechanism on the close relationship between the abnormal neural oscillations and synchrony and the core symptoms of schizophrenia.

## Abbreviations

CLGF: Coupled local and global feedback; PLV: Phase locking value; FB: Feedback.

## Competing interests

The authors declared that they have no competing interests.

## Authors’ contributions

KHC designed the project and supervised the research, JSK (Jun Soo Kwon) designed and managed the clinical study, KN and KHC performed the mathematical modeling and MEG analysis, DS assisted mathematical modeling, KSS, JSK (June Sic Kim), and CKC has designed the resting paradigm, JYH, JHJ and JSK (Jun Soo Kwon) recruited the patients and performed the clinical assessments, and KN, KSS, DS, and KHC wrote the manuscript. All authors read and approved the final manuscript.

## Supplementary Material

Additional file 1 Supporting figures. Click here for file

Additional file 2 Supporting methods & references. Click here for file
