# Supplementary material for: Impaired coupling of local and global functional feedbacks underlies abnormal synchronization and negative symptoms of schizophrenia
Source: BMC Syst Biol. 2013 Apr 10;7:30. doi: 10.1186/1752-0509-7-30 (PMC3639871; doi:10.1186/1752-0509-7-30)
Supplement: Additional file 1 — Supporting figures. [file 1752-0509-7-30-S1.pdf]

Impaired Coupling of Local and Global Functional Feedbacks Underlies Abnormal Synchronization and Negative Symptoms of Schizophrenia

Kyungchul Noh[1], Kyung Soon Shin[2], Dongkwan Shin[1], Jae Yeon Hwang[4,6], June Sic Kim[3], Joon Hwan Jang[5], Chun Kee Chung[3], Jun Soo Kwon[2,5,6†], and Kwang-Hyun Cho[1*]

[1]Department of Bio and Brain Engineering, Korea Advanced Institute of Science and Technology (KAIST), Daejeon, Republic of Korea

[2]Clinical Cognitive Neuroscience Center, Neuroscience Institute, SNU-MRC, Seoul, Republic of Korea

[3]MEG Center, Department of Neurosurgery, Seoul National University College of Medicine, Seoul, Republic of Korea

[4]Department of Psychiatry, SMG-SNU Boramae Medical Center, Seoul, Republic of Korea

[5]Department of Psychiatry, Seoul National University College of Medicine, Seoul, Republic of Korea

[6]Department of Brain and Cognitive Sciences – World Class University Program, College of Natural Sciences, Seoul National University, Seoul, Republic of Korea

Supporting Figures

[†] Co-corresponding author, E-mail: kwonjs@snu.ac.kr, Phone: +82-2-2072-2972, Fax: +82-2-747-9063.

[*] Corresponding author, E-mail: ckh@kaist.ac.kr, Phone: +82-42-350-4325, Fax: +82-42-350-4310.

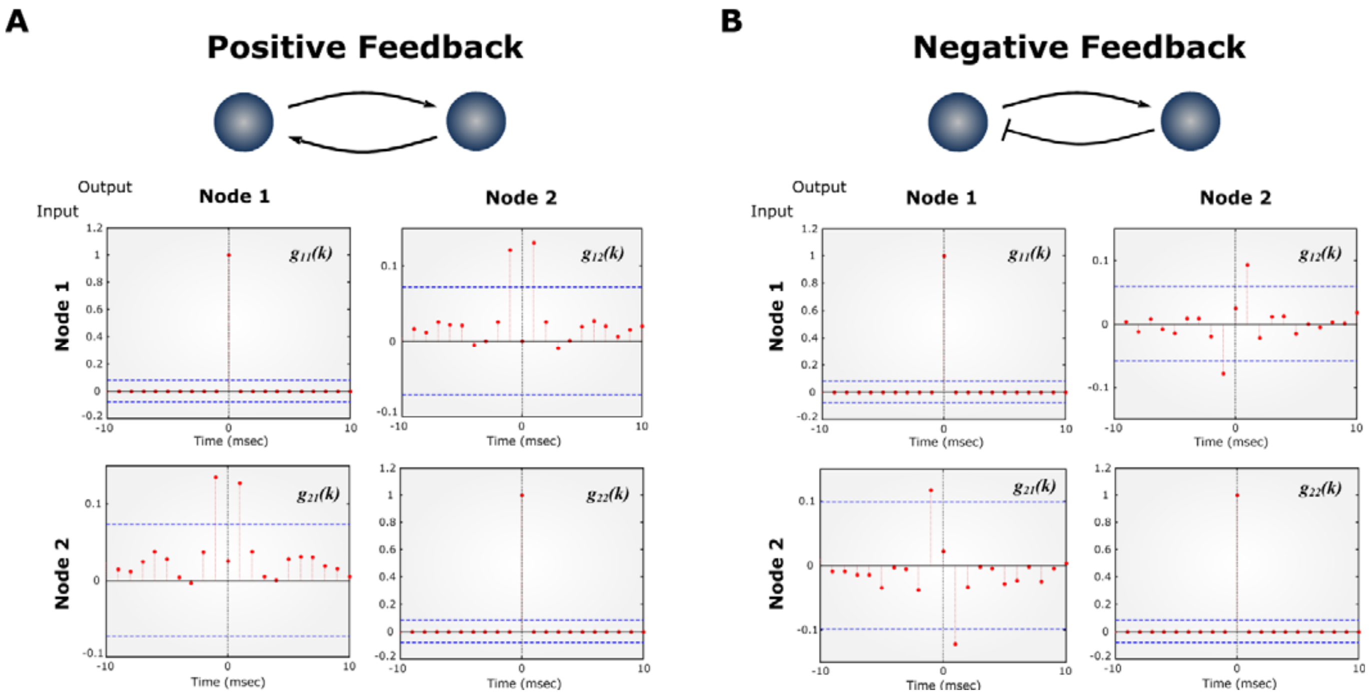

Figure S1. The impulse responses used for identification of (A) positive and (B) negative feedback loops. In both figures, the row index denotes input signals and the column index indicates output signals. The non-causal (causal) components are the values that lie on the negative (positive, respectively) part of time axis. Both causal and non-causal impulse responses $g_{11}(k)$, $g_{12}(k)$, $g_{21}(k)$, and $g_{22}(k)$ are represented by red dots. The blue dashed lines indicate 95% confidence interval, which indicates significant impulse response components outside this interval. The concurrences of the significant non-causal components of $g_{12}(k)$ and $g_{21}(k)$ reflect the existence of a feedback loop. (A) Both positive signs of significant causal components indicate that the identified feedback loop is a positive feedback loop, and (B) the opposite signs of significant causal components of $g_{12}(k)$ and $g_{21}(k)$ indicate that it is a negative feedback loop.

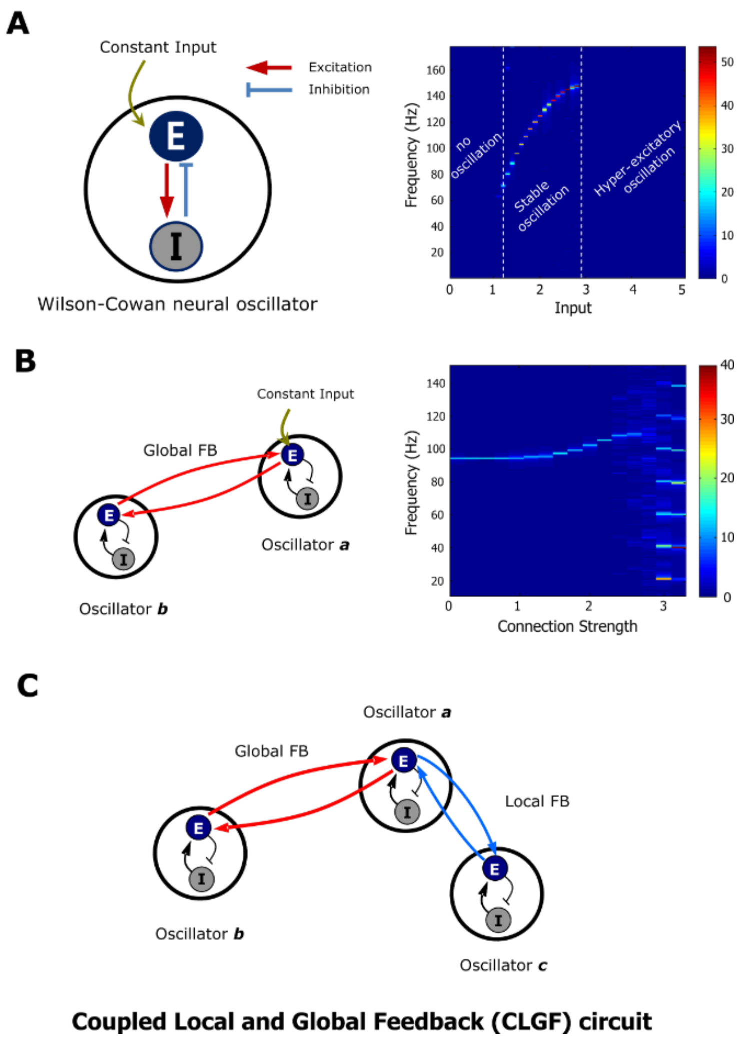

Figure S2. The simulation of the CLGF circuit. (A) (*Left*) Description of the Wilson-Cowan neural oscillator. The oscillator is composed of excitatory and inhibitory neuronal pools. A constant external input is given to the excitatory neuronal pool in the single oscillator. (*Right*) There are three regimes of the output characteristics with varying input values: no oscillation, stable oscillation, and hyper-excitatory oscillation. Stable oscillations were shown only in the input range of 1.2 to 3. (B) (*Left*) Within the regime of stable oscillation in the oscillator *a*, excitatory pools of both oscillators *a* and *b* were connected with each other by the global positive feedback connection. (*Right*) The dominant frequency was abruptly changed into beta band for strong connections. Finally, we connected the excitatory pools of both oscillators *a* and *c* with the local positive feedback to form the CLGF circuit. (C) Description of the CLGF circuit composed of three Wilson-Cowan neural oscillators, *a*, *b*, and *c*. Both oscillators *a* and *b* are connected by the global positive feedback loop and the oscillator *c* is connected to the oscillator *a* through the local positive feedback loop. The global and local positive feedbacks are distinguished by the time delay of each feedback.

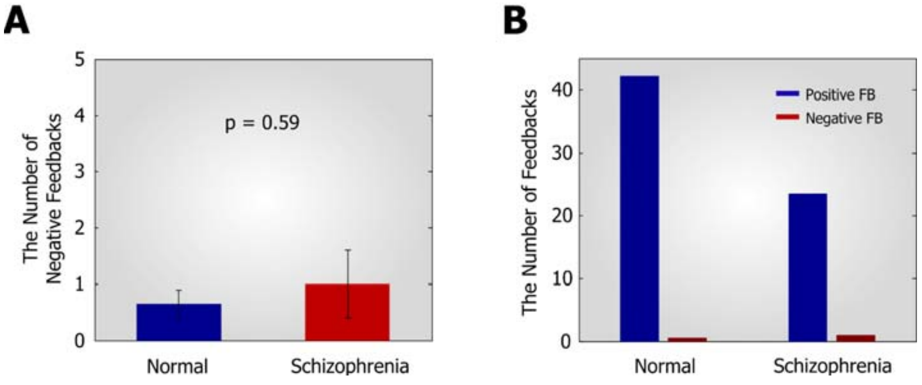

Figure S3. Comparison of the number of feedback connections between normal and patient groups. (A) The number of negative feedbacks was not significantly different between the two groups (p = 0.59). (B) In both groups, the number of negative feedbacks was much smaller than that of positive feedbacks. The bars in (A) and (B) denote means, and the vertical lines in (A) represent standard errors.

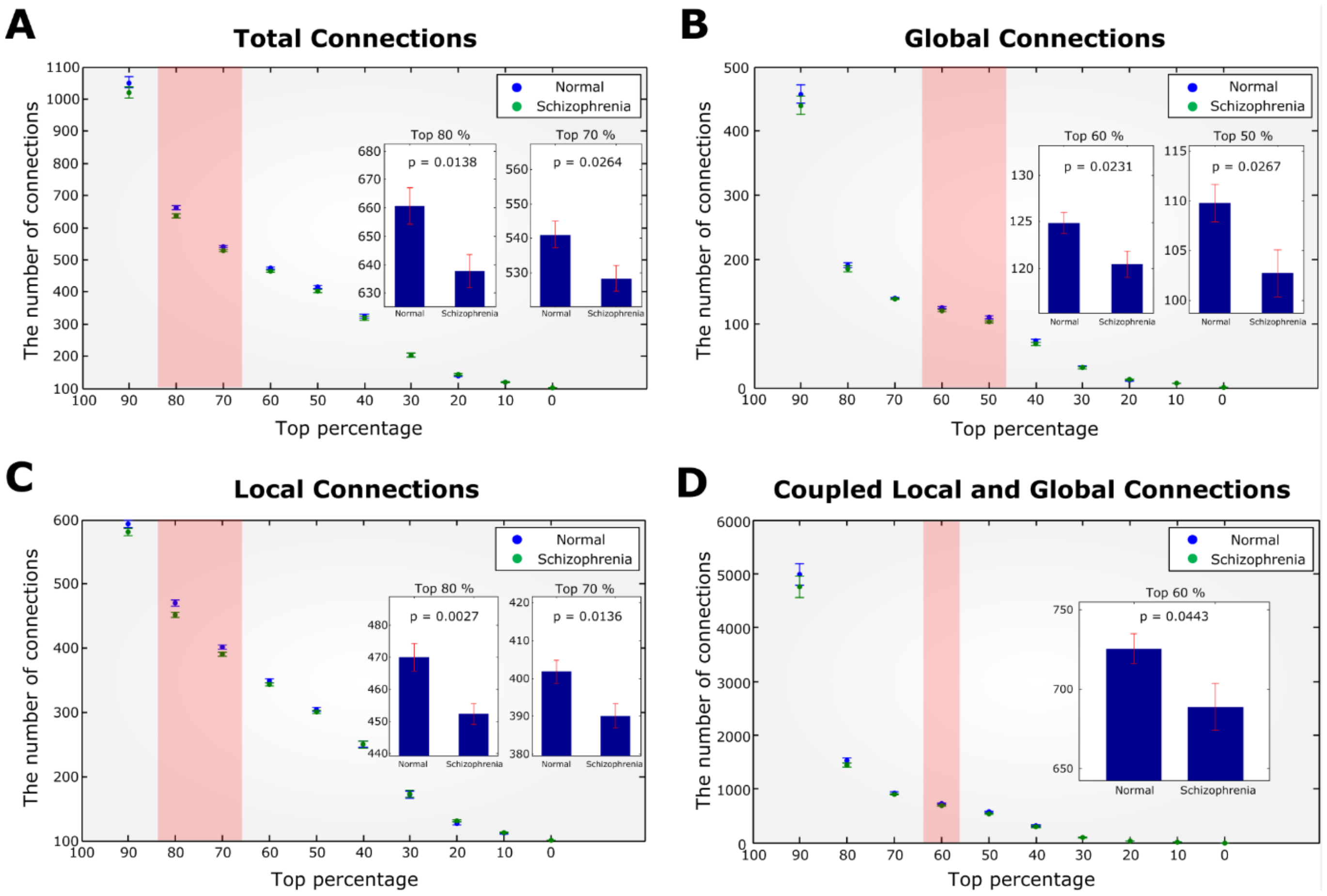

Figure S4. The functional connectivities obtained from partial correlation analysis. The numbers of (A) total, (B) global, (C) local, and (D) coupled local and global connections between normal (blue) and patient (green) groups are shown. For each type of connections, we found that the number of connections was significantly reduced in the schizophrenia patients group at certain top percentages of partial correlation as denoted by red-shaded boxes and bar graphs (insets) for statistical significance between the two groups. The *x*-axis represents the top percentage of partial correlation values and *y*-axis the number of connections. Blue and green dots denote mean values, and vertical lines represent standard errors. In each inset, bars and vertical lines represent mean values and standard errors, respectively.

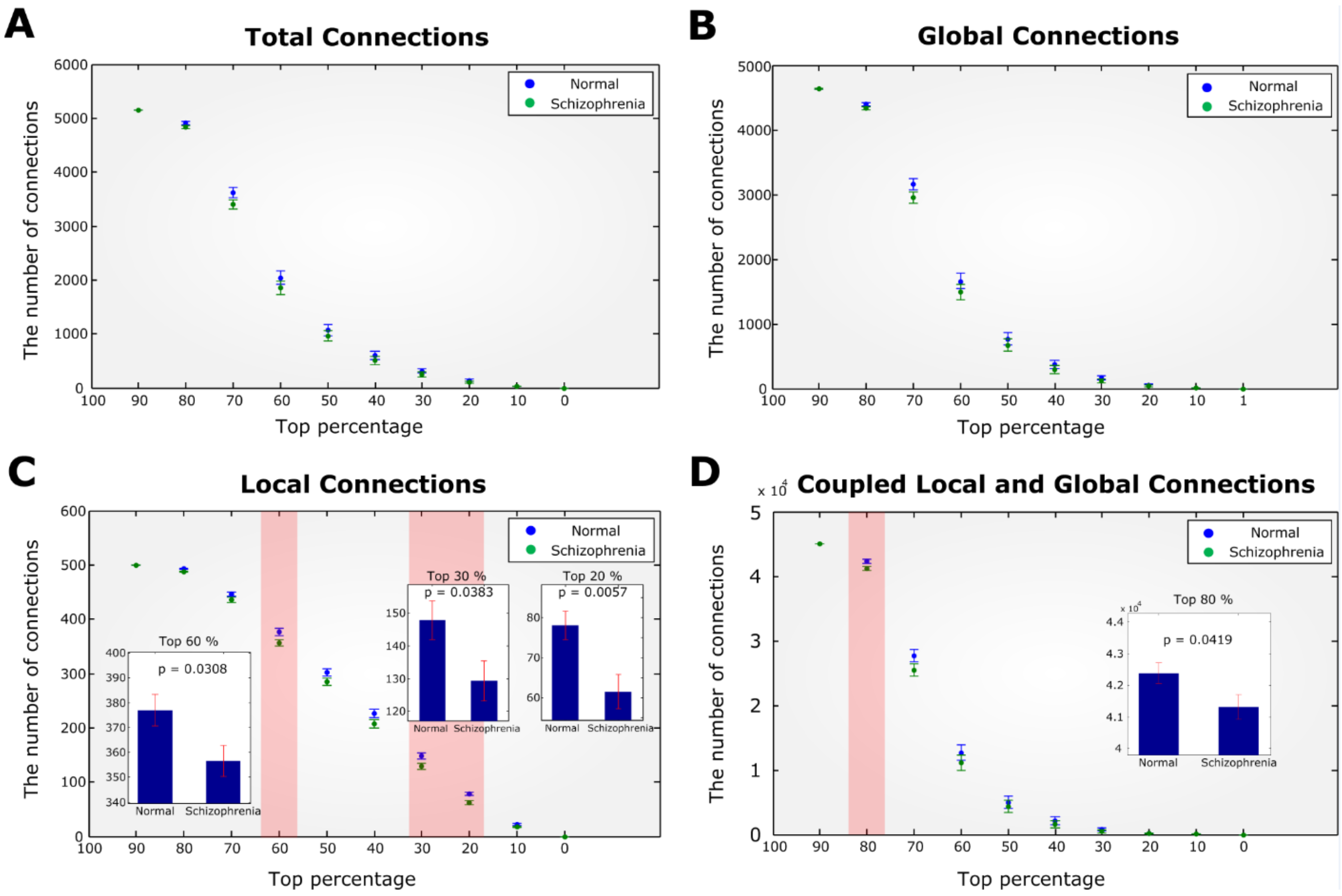

Figure S5. The functional connectivities obtained from mutual information (MI) analysis. The numbers of (A) total, (B) global, (C) local, and (D) coupled local and global connections between normal (blue) and patient (green) groups are shown. We found that the number of connections was significantly reduced in the schizophrenia patients groups at certain top percentages of MI values in local (C) and coupled local and global (D) connections as denoted by red-shaded boxes and bar graphs for statistical significance between the two groups. All notations are the same as those of Figure S4.

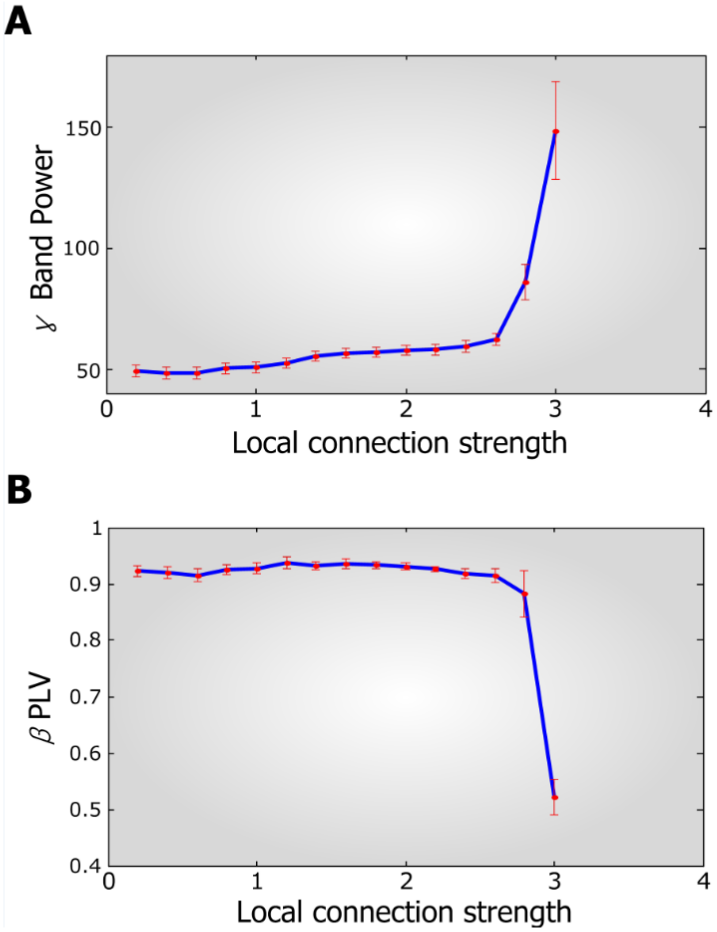

Figure S6. Changes of the gamma power and beta PLV for the CLGF circuit along with the increase of local connection strength with various time delays. Global and local time delays were randomly selected in the ranges of 15 ~ 25 ms and 6 ~ 10 ms, respectively. 50 trials of simulations were averaged for each local connection strength where hyper-excitatory oscillations were excluded. (A) The gamma band power was abruptly increased beyond a threshold of local connection strength. (B) On the other hand, beta band phase synchronization was suddenly decreased beyond the threshold. The vertical lines represent standard errors.
