# Supplementary material for: Impaired coupling of local and global functional feedbacks underlies abnormal synchronization and negative symptoms of schizophrenia
Source: BMC Syst Biol. 2013 Apr 10;7:30. doi: 10.1186/1752-0509-7-30 (PMC3639871; doi:10.1186/1752-0509-7-30)
Supplement: Additional file 2 — Supporting methods & references. [file 1752-0509-7-30-S2.pdf]

Impaired Coupling of Local and Global Functional Feedbacks Underlies Abnormal Synchronization and Negative Symptoms of Schizophrenia

Kyungchul Noh[1], Kyung Soon Shin[2], Dongkwan Shin[1], Jae Yeon Hwang[4,6], June Sic Kim[3], Joon Hwan Jang[5], Chun Kee Chung[3], Jun Soo Kwon[2,5,6†], and Kwang-Hyun Cho[1*]

[1]Department of Bio and Brain Engineering, Korea Advanced Institute of Science and Technology (KAIST), Daejeon, Republic of Korea

[2]Clinical Cognitive Neuroscience Center, Neuroscience Institute, SNU-MRC, Seoul, Republic of Korea

[3]MEG Center, Department of Neurosurgery, Seoul National University College of Medicine, Seoul, Republic of Korea

[4]Department of Psychiatry, SMG-SNU Boramae Medical Center, Seoul, Republic of Korea

[5]Department of Psychiatry, Seoul National University College of Medicine, Seoul, Republic of Korea

[6]Department of Brain and Cognitive Sciences – World Class University Program, College of Natural Sciences, Seoul National University, Seoul, Republic of Korea

Supporting Methods & Reference

[†] Co-corresponding author, E-mail: kwonjs@snu.ac.kr, Phone: +82-2-2072-2972, Fax: +82-2-747-9063.

[*] Corresponding author, E-mail: ckh@kaist.ac.kr, Phone: +82-42-350-4325, Fax: +82-42-350-4310.

Supporting Methods

1. Partial correlation

Pearson correlation is widely used as an index of the dependence of the time series of two sensor locations in the EEG or MEG. The correlation between two sensors represented by Pearson correlation might also be affected by the indirect relationship caused by sequential pathways or common sources. To resolve such a problem, partial correlation can be employed and used to prevent a possible false prediction about direct links between two sensors [1]. The main purpose of using the partial correlation is to avoid predicting non-direct functional links.

The partial correlation between node *i* and *j*, $r_{ij|k}$ is defined as

$$r_{ij|k} = \left| \frac{r_{ij} - r_{ik} r_{jk}}{\sqrt{(1 - r_{ik}^2)(1 - r_{jk}^2)}} \right|$$

where r_{ij} is the absolute value of Pearson correlation between *i* and *j*. Since both negative and positive correlation values indicate the strength of functional connections, we considered the absolute values of correlations which range from 0 to 1 corresponding to 'no' and 'perfect' correlation, respectively. We calculated the partial correlation in consideration of all possible choices of single *k* node (except *i* and *j*) and then selected the minimum among the obtained values.

To create the association matrix between 102 MEG magnetometer sensors, 20 *sec* time window was used and the partial correlation values were calculated in four windows that were not overlapped with each other (4 × 20 *sec* = 80 *sec*) for each subject in a group. For each individual subject, four association matrixes were averaged, and we counted the number of each type of connections (total, global, local, and coupled local and global connections) whose values of partial correlation are ranked in the top *p*-percentage. The same procedure was repetitively applied to all subjects in the two groups. A two-tailed *t*-test was carried out to test the group differences and α-level was set to 0.05 for all statistical tests.

2. Mutual Information

Mutual information (MI) measures the mutual dependence or information gained about one signal from another. It represents the amount of information about X_i that X_j contains. If X_i is independent of X_j, then MI is zero, but the higher the MI between two signals, the more information they contain about each other. MI is widely used to construct a functional network in EEG or MEG studies. We calculated MI values between 102 MEG magnetometer sensors using the following equation:

$$MI_{ij} = MI_{ji} = -\sum_{X_i(t),X_j(t)} P(X_i(t), X_j(t)) \log_2 \frac{P(X_i(t), X_j(t))}{P(X_i(t)) \cdot P(X_j(t))}$$

where $P(X_i(t), X_j(t))$ is the joint probability density function (PDF) between two time series $X_i(t)$ and $X_j(t)$. MI is a symmetric function implying $MI_{ij} = MI_{ji}$. If $X_i(t)$ is independent of $X_j(t)$, then MI is zero. On the other hand, the higher the MI between two signals, the more information they contain about each other. Subsequent procedures were identical with those of partial correlation as mentioned above. All functional network estimations were conducted using MATLAB 7.8.0 (R2009a) in Windows.

Reference

1. Jalili M, Knyazeva MG: **Constructing brain functional networks from EEG: partial and unpartial correlations.** *J Integr Neurosci* 2011, **10**:213-232.
